# The Properties of Terrestrial Laser System Intensity for Measuring Leaf Geometries: A Case Study with Conference Pear Trees (*Pyrus Communis*)

**DOI:** 10.3390/s110201657

**Published:** 2011-01-28

**Authors:** Mathilde A.F. Balduzzi, Dimitry Van der Zande, Jan Stuckens, Willem W. Verstraeten, Pol Coppin

**Affiliations:** 1 Biosystem Department, Katholieke Universiteit Leuven, M3-BIORES Willem de Croylaan 34, BE-3001 Leuven, Belgium; 2 Department VI of the Royal, Management Unit of the North Sea Mathematical Model (MUMM), Belgium Institute of Natural Science, Rue Vautier, 1000 Brussel, Belgium; 3 Royal Netherlands Meteorological Institute, Climate Observations, PO Box 201, NL-3730 AE, De Bilt, The Netherlands; 4 Applied Physics, Eindhoven University of Technology, PO Box 513, 5600 MB, Eindhoven, The Netherlands; 5 GeoID, Researchpark Haasrode, Interleuvenlaan 62, 3001 Leuven, Belgium

**Keywords:** leaf inclination, leaf geometries, intensity return, TLS, Ghost point, mesh, conference pear tree

## Abstract

Light Detection and Ranging (LiDAR) technology can be a valuable tool for describing and quantifying vegetation structure. However, because of their size, extraction of leaf geometries remains complicated. In this study, the intensity data produced by the Terrestrial Laser System (TLS) FARO LS880 is corrected for the distance effect and its relationship with the angle of incidence between the laser beam and the surface of the leaf of a Conference Pear tree (*Pyrus Commmunis*) is established. The results demonstrate that with only intensity, this relationship has a potential for determining the angle of incidence with the leaves surface with a precision of ±5° for an angle of incidence smaller than 60°, whereas it is more variable for an angle of incidence larger than 60°. It appears that TLS beam footprint, leaf curvatures and leaf wrinkles have an impact on the relationship between intensity and angle of incidence, though, this analysis shows that the intensity of scanned leaves has a potential to eliminate ghost points and to improve their meshing.

## 1. Introduction

Generally, the canopy represents the interface where most of the fundamental interactions between vegetation and atmosphere take place [1]. The canopy governs for example radiation interception which is the driving force for photosynthesis and controls growth and production [2–4]. Since energy and material exchanges in canopies occur primarily across leaf surfaces, it is an incentive to develop measurement techniques that are able to derive details at the leaf level. Leaves have a temporal and spatial organization which includes their position, dimension, quantity, type, and connectivity with other canopy elements of the above-ground vegetation [5]. This is what is generally equated as canopy structure.

An important index to describe vegetation structure is the Leaf Area Index (LAI) which is used in any flux transfer study as gases exchange e.g., CO_2_ [6] or radiative transfer [7]. With respect to the radiation interception, LAI is defined as the total one sided leaf area per unit ground surface area [8]. However, in [9], the authors proposed an alternative definition of LAI that takes into account curvatures, wrinkles and leaf elevation.

Leaf inclination (elevation, roll and azimuth) affects the photosynthesis process in two ways: (i) it provides a mechanism for the plant to achieve favorable photosynthetic rates at specific times during the day, and (ii) it limits the impact of high incidence photon irradiance unfavorable for photosynthesis [10]. A more general index that describes leaf inclination is the Leaf Angle Distribution (LAD). It is an essential parameter for characterizing canopy structure and plays a crucial role in the simulation of radiative transfer [11]. In such studies, canopies are represented either as a turbid medium or as discrete scatterers [12,13]. However, in the case of modeling the radiative transfer of trees, a detailed tree description is more relevant. For instance, leaves’ elevations are generally not randomly distributed but are directly linked to their position in the tree [14,15]. Working with reconstructed virtual trees [16] and/or with accurate descriptions of leaf curvature would enable more accurate and geometrically explicit simulations for flux transfer studies and for simulation of radiative transfer in the canopy.

Several innovative remote sensing methods attempted to describe vegetation structure parameters such as LAI or LAD in a fast, repeatable and accurate way. The use of photographs [17], light sensors [18], and tele-lenses [19] offers possible solutions for the structure assessment problems but mostly encounters practical problems in field conditions. Light Detection And Ranging (LiDAR) technology potentially provides a novel tool for generating an accurate and comprehensive 3D mathematical description of tree and canopy structure. This remote sensing technique gathers structure information by scanning objects in a non-destructive manner and without physical contact [20]. Unlike passive systems such as hyperspectral scanners, which need an independent energy source (*i.e*., the sun), the active Terrestrial LiDAR System (TLS) carries its own energy source. TLSs use powerful highly collimated optical light or laser light as sensing carrier. The energy of such a laser beam interacting with the measured object is partially reflected back in the direction of the laser system where it is registered by a sensor and used to measure the distance between this sensor and the illuminated spot on the measured object. This measurement can be achieved by detecting the Time Of Flight (TOF), by measuring the phase shift of an Amplitude Modulated Continuous Wave (AM-CW) or by using a Frequency Modulated Continuous Wave (FM-CW) technology [21]. By providing a 3D image of its surrounding scene, TLS became a common tool in archeology, architecture and topography (e.g., [22]), but also in agriculture and forestry. In forestry, TLS has been used to determine forest metrics such as the diameter at breast height (DBH), tree height, stem density, volume estimation, gap fraction, LAI and vertical plant profile [23–25,20]. In agriculture, this device has been used to estimate the vegetative volume and its surface area [26,27].

However, to get an accurate and precise description of the geometry of a small object as eg. a leaf, the number and density of the point cloud is determinant. In the case of scanned foliage, the scan quality could decrease because of:

- The shadow effect. The leaves on the TLS field of view foreground hide leaves on the background. Those are either partially scanned or not scanned at all [33]. Thus, the number of point per leaves is reduced.
- The wind which may move the branches and the leaves during the scan process and decrease the quality of the scan.
- The leaves reflectance, the geometric calibration of the TLS, the foliage distance and the TLS beam angle of incidence with the leave surface [34–36] which could reduce the precision of the scanning.
- The fact that lasers are spherical range finders. That means that the distance between two points on a flat surface will increase with the distance to the beam aperture [20]
- The light ambiance for large distance [29] as it avoid the sensor to record low reflectance.
- The ratios between the TLS beam footprint and the size of the scanned object, e.g., leaves [37]. This footprint diameter depends on the TLS beam incidence angle with the leaves surface, the distance and the device features. The TLS beam footprint could overlap the scanned object. In this case, the point cloud appears more like a set of ghost points or mixed pixels [38] and does not represent the object accurately and/or with precision.

In conclusion, the point density for the foliage could be too sparse to provide detailed information to derive leaf inclination and other geometric information such as area, shape or inclination. Traditionally, leaf inclination is directly determined with a protractor [39,40] or with an electromagnetic digitizer [41,42]. Those two methods are time consuming and labor intensive. That is the reason why the TLS became a new opportunity in foliage studies. For instance, allometry and transpiration studies with TLS use a point cloud voxelisation technique [43,44]. Only a few studies have managed to get individual leaf geometries such as inclination and/or leaf area. This has been done by Hosoi *et al.* [45] for wheat leaf elevation distribution and by Chambelland *et al.* [46] for young beech leaf geometries using a Konica VIVID 910, a very high resolution and precise scanner (approximately 0.16 mm). In this latest article, authors work on single leave scans, at close range (<2.5 m), with an angle of incidence close to 0° and under laboratory conditions. Thereby, the authors do not encounter the issues mentioned above. In this study, we would like to improve the point cloud meshing process for *in situ* scanned tree with lower precision scanners as the FARO LS880 can be. In order to do so, the first step would be to assess the point cloud quality to determine leaf geometries. As recent TLSs provide an intensity value, the idea of this research is to investigate the potential of this intensity to improve correction of the point cloud and its meshing. This one is function to the scanned object distance to the beam aperture, the angle of incidence between the beam and its surface and optical properties of the scanned material ([28–30] and [56]), e.g., leaves. To avoid the distance effect, the TLS intensity could be corrected [31]. It is necessary since only a corrected intensity can be used to establish a consistent relationship between intensity and angle of incidence between the leaf surface and the TLS beam and this, for any distance. Once this latest relationship is known, then the intensity can be used as an additional indicator for determining the quality of the point cloud (ghost point) and for improving correction and meshing methods on scanned leaves.

## 2. Methods and Materials

### 2.1. Terrestrial LiDAR System (TLS)

#### 2.1.1. System Characteristics

The TLS FARO LS880 is used in this study. The rotation of a mirror placed at 45° to the laser beam aperture (horizontal rotation) and the rotation of its trunnion (vertical rotation) provide a panoramic view of the scene that is surrounding the TLS as a 3D point cloud in a Cartesian or in a spherical basis. The scans are proceeded with an angular resolution of 0.018° for both azimuthal and elevation rotation. This device uses the AM-CW technology: the amplitude of the laser is modulated and an analysis of the frequencies of the backscattered signal provides the distance. Between the mirror and the photodiode of the scanner, optical elements (e.g., filters) reduce the intensity for small distances to avoid overexposure of the sensor. Therefore, the relationship between the intensity and distance follows neither the inverse square power law nor any linear function. In addition, the electric-converted signal passes through a logarithmic amplifier that provides a logarithmic relationship between different reflectance [28]. Each point has an extra dimensionless value that is the intensity (ranging from 0 to 2047 in digital numbers) measured by the system. Details on the features of the TLS FARO LS 880 are given Table 1.

#### 2.1.2. TLS Intensity and Its Dependencies

Theoretically, the photometric appearance of an object depends on surface geometry, material properties, illumination and viewing direction of the camera (*i.e*., the TLS sensor) [47]. With the geometric property of a collimated laser beam and the emitter and sensor diameter, the relationship between the emitted power (P_T_) and the received power (P_R_) is highly dependent on angle of incidence, on distance and on material reflectance properties [32]:

(1) $$P_{R}=\eta .\frac{A_{R}.P_{T}.\rho (\omega ).\cos(\omega )}{\pi .d^{2}}$$

with A_R_ the receiver aperture area, η the receiver’s efficiency, ω the angle of incidence with the material, ρ(ω) the reflectance value in function the angle of incidence between the TLS beam and the material surface (constant in the case of Lambertian material) and d the distance between the TLS beam aperture and the scanned object. As the TLS FARO LS880 has an intensity filter and with the assumption that this filter has only an impact on the intensity variations due to distance, the inverse square law could be replaced by a device specific distance function f. Finally, the intensity is modified by a logarithmic converter. The received power could be expressed as follows:

(2) $$P_{R}=\text{log}\left(\eta .\frac{A_{R}.P_{T}.\rho (\omega ).\cos(\omega )}{\pi }.f(d)\right)$$

(3) $$=\underset{\left(a\right)}{\underset{︸}{\text{log}(\eta )+\text{log}(A_{R})+\text{log}(P_{T})-\text{log}(\pi )}}+\underset{\left(b_{1}\right)}{\underset{︸}{\text{log}(\rho (\omega ))}}+\underset{\left(b_{2}\right)}{\underset{︸}{\text{log}(\cos(\omega ))}}+\underset{\left(b_{3}\right)}{\underset{︸}{\text{log}(f(d))}}$$

where (a) is a constant term of the formula while (b_1_), (b_2_) and (b_3_) are its variable terms. Expressed through a logarithmic function, the nature of the intensity, distance and angular dependencies changes:

- there is a vertical translation of the graphs representing the received power and distance relationship at a fixed angle of incidence appears and this, for two different material reflectances (b_1_ and b_3_). This is due to the logarithmic product-to-sum reduction.
- With the same reasoning, the received power and angle of incidence relationship has the same shape through distance (b_2_ and b_3_).

In [28–30] and [56], the authors experiment with the influence of distance, material and angle of incidence on the intensity on a Spectralon® and retrieve those two properties. In this publication, we will consider the received power as the intensity recorded by the TLS.

As the objects of our study are leaves, the diameter of the TLS beam footprint is an important parameter. A flat surface with an angle of incidence of ω, which has its center at a distance d from the TLS beam aperture and with a TLS beam radius of r and a divergence of δ, one gets the footprint major axis:

(4) $$\frac{2.r}{\cos(\omega )}+\left(\frac{d+r.\text{tan}(\omega )}{\cos(\omega -\delta )}+\frac{d-r.\text{tan}(\omega )}{\cos(\omega +\delta )}\right).\sin(\delta )$$

In the case that the TLS footprint size is too large compared to the scanned object dimensions, a crosstalk effect and a mixing of the intensity and distance in the point cloud occur [38].

According to the manufacturer, ambient light (e.g., sun) has little impact on the intensity. It does not fade out the signal and the intensity data are similar for scanned scene with different ambient light. However, there is more noise in the point cloud with increasing distance and sometimes even no data at all, especially for low intensity. The FARO LS880 has been designed to be insensitive to solar irradiance, at least for ranges smaller than 10 m and/or for surfaces with medium to high reflectance.

### 2.2. Measurement Setup

#### 2.2.1. Study of the distance effect on the TLS intensity

As discussed in Section 2.1.2., the relationship graph between the intensity as the received power and the distance follows the property of a vertical translation for different material reflectances (Equation 3.b1.). In [28], this relationship is more variable for close distance (<3 m) due to the filter in the front of the TLS sensor whereas they resemble the inverse square law of light intensity for greater distances. Thus, to avoid the distance effect as in [31], materials with different reflectances are scanned at varying distances. Its aim is to retrieve this vertical translation of the relationship graph. For this publication, materials are scanned on a board perpendicular to the laser beam at 0.35, 0.45, 0.6, 0.75, 0.8, 1, 1.5, 1.85, 2.15, 2.6, 3.6, 5, 7.5 and 10 m. Materials reflectance properties are measured at 785 nm with a Spectra-Vista HR1024 spectroradiometer (spectral resolution of 3.5 nm between 350 and 1,000 nm). Measured materials are a 99% reflectance Spectralon® whitepanel, five mate Canson® drawing papers (with 3%, 68%, 48%, 80%, 83% reflectance), a 22% reflectance Kodak® Grey Card and a matte 3% reflectance painted board. All these materials are either Lambertian or matte to be able to neglect the intensity variation due to the geometry of the measurement (combination of a hemispherical scan with flat materials), especially for short distances. Each material has a size of at least 8 x 8 cm to avoid intensity mixing due to the TLS beam footprint size at the point representing the scanned surface at 10 m. Finally, a piecewise interpolation of the intensity in function of the distance is calculated to retrieve intensity values at intermediate distances. This measurement setup allows a distance correction of the intensity.

#### 2.2.2. Setting the Relationship between the Beam Angle of Incidence with a Leaf Surface and the Corrected Intensity

**Figure 1.**
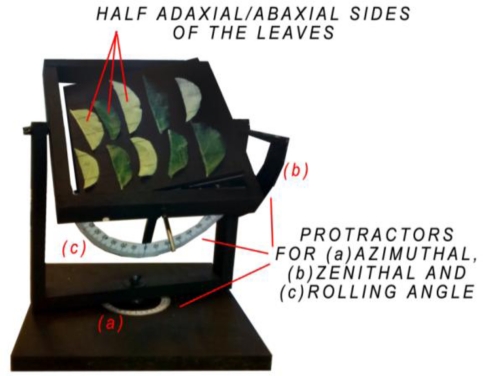
The goniometric platform with its (**a**) azimuth; (**b**) elevation and (**c**) rolling angle protractors. To be flattened, half adaxial and abaxial leaf faces are fixed with black strings.

In [30] and [56], authors shows the relationship between the angle of incidence and the intensity for a 99%-Spectralon®. To study the influence of the angle of incidence on the intensity with materials such as leaves, a goniometric platform has been built (Figure 1). It allows complete rotations around its vertical (azimuth) and horizontal axes (elevation). Protractors are fixed onto the structure of the goniometric platform to show the azimuth/elevation and rolling angle. The rolling angle variation is not used in this study. The platform is painted with a 3%-reflectance black paint for an easier segmentation of the point cloud.

Ten leaves were randomly picked from 30 2-year old Conference pear trees on June 16th 2010. Those trees are planted in two rows of 15 trees in an East-West direction with a distance of 30 cm between the trees and 360 cm between the rows. They are located in Heverlee, Belgium (50°51′33.89″N, 4°40′48.45″E). Since the leaves are curled [48], they are cut in two parts along their central vein to be flattened as much as possible before the scanning procedure. They are fixed (abaxial and adaxial side) on the goniometric platform with black painted strings (Figure 1) and are scanned within the hour after collection. The average length of those leaves is 6.47 cm (0.5 cm standard deviation) and their average (half)-width is 2.19 cm (0.38 cm standard deviation). The goniometric platform is placed at 2.16 m from the TLS beam aperture. An increment of 20° on the vertical axis and subsequently on the horizontal axis is applied for each scan to get an angle of incidence ranging from 0° (perpendicular to the laser beam) to 80° (almost parallel to the laser beam). For each leaf, one to three sub-selections are extracted from the point cloud depending on the angle of incidence and the size of the leaf. The angle of incidence with the leaf surface is then compared to the averaged corrected intensity on those sub-selections. The relationship between the intensity and the angle of incidence with pear tree leaves is so deducted.

**Figure 2.**
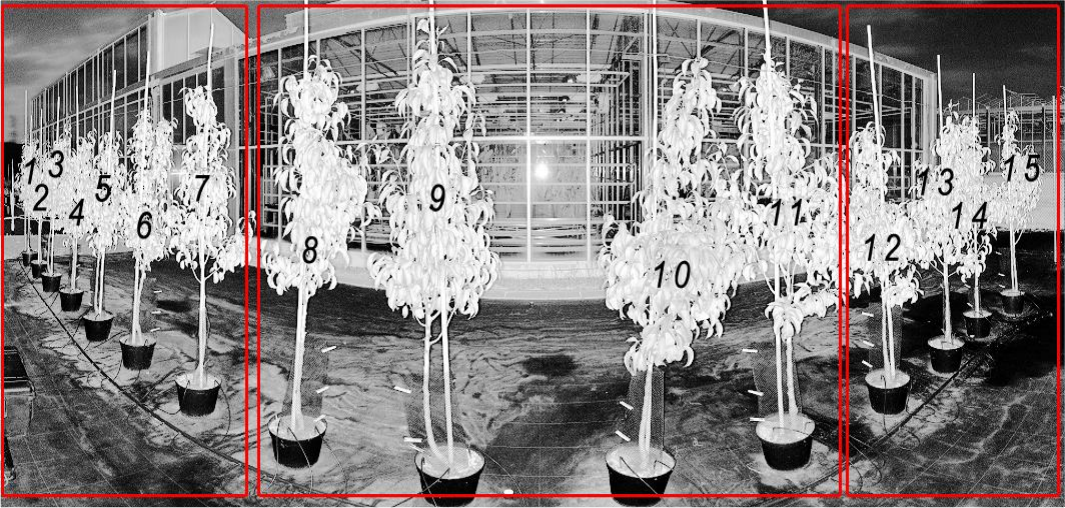
Part of a hemispherical projection of a TLS scan of 15 two years old pear trees (first row). The corrected intensity and angle of incidence relationship is tested on leaves of those trees. Trees are grouped by their distance to the beam aperture (red frames).

In a second experiment, from each of the 15 pear trees of the first row (Figure 2) a second scan is made on June 24th 2010. A sub-selection of a flat part of each scanned leaf is made. The average corrected intensity of this sub-selection is then related to the angle of incidence provided by a Least Square Regression (LSR). This relationship is then compared to the previously established intensity and angle of incidence relationship. Compared to the latter experiment, leaves are not at a constant distance and are not flattened. Finally, the LSR is made on seven entire leaves for tree n°9 (Figure 2) to gain a more thorough understanding of the leaves geometry impact on the TLS point cloud and intensity data.

In this case, ghost points as wrinkles and curvatures are also selected. Then, the differences (Δω) between the angle of incidence found by this LSR and the angle of incidence provided by the intensity for each hit of the TLS beam are mapped for each of those seven leaves:

(5) $$\Delta \omega (x)=\omega_{I}(x)-\omega_{\text{LSR}}$$

with ω_I_(x), the angle of incidence computed with the intensity and angle of incidence relationship at a point x on the scanned leaf and ω_LSR_ the angle of incidence provided by the LSR on the entire leaf. The distributions (with normalized quantities) of those Δω values are shown. The difference between the angle of incidence provided by the LSR and the one deduced by the average of the corrected intensity on the leaf is calculated.

### 2.3. TLS Data Preprocessing and Analysis

**Figure 3.**
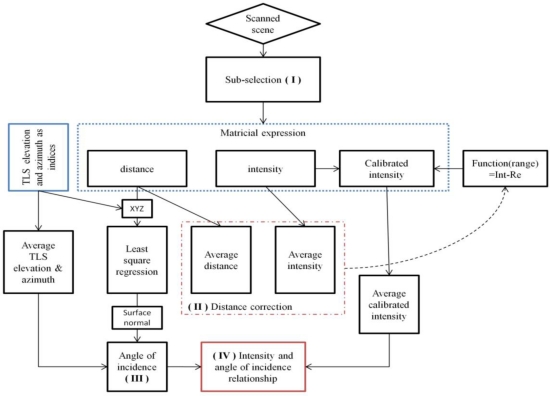
Analysis flowchart: (I) A semi-automatic and manual selection in the point cloud is proceeded. It takes into account a distance and an intensity threshold to limit unwanted point as ghost point or leaf curvature. (II) The average distance and average intensity are calculated from the selected point cloud. Their relationship is used to correct the distance effect by replacing the intensity value by a reference value (correction of the distance effect on the intensity). (III) The angle of incidence with the selected surface is calculated thanks to a LSR. (IV) The corrected intensities values of the selected points are averaged. The angle of incidence is then related to this averaged corrected intensity.

First, a manual sub-selection of the point cloud and their corresponding intensity is made. A second sub-selection is made based on an intensity and distance threshold [Figure 3(I)]. To do so, a reference point is selected and neighboring points are considered hit points if their intensity and distance from the selected point are within the thresholds values. This approach limits the selection of edge effects of leaves as well as the distance and intensity crosstalk effect, mixing of multiple objects within the beam footprint [49,38]. It also limits the selection of curvature and wrinkles of leaves. After data extraction, the analysis gives distance values, angle of incidence provided by the LSR, the average and standard deviation on the selected point’s intensity and finally, the number of selected points.

#### 2.3.1. Correction of the Distance Effect on the Intensity

A first study is made to establish the relationship between the intensity and the distance with the set up described in Section 2.2.1. In [28], the relationship graph between intensity and distance for the FARO LS880 presents a vertical translation for the different reflectance. In this case, the relationship between intensity and distance can be interpolated by the same polynomial. In addition to the method suggested in [31], where the intensity is normalized by a 99%-Spectralon® intensity, an interpolation of the data is calculated to get intermediate values of intensity in function of the distance. As the relationship does not follow any analytical function because of the intensity filter (Equation 3), piecewise polynomial interpolations of order one or two are calculated and as those interpolations are equal, but vertically translated (Equations 3 and 4), only one piecewise polynomial interpolation should be calculated, namely, the one of the 99%-Spectralon® (denoted as f_99%_).

Once done, a constant value c for a target material at a given ω is determined by the difference between its intensity value and the intensity value of the 99%-Spectralon® at a fixed reference distance d_ref_. One has c_ref_ = f_99%_(d_ref_) − I (d_ref_) with I (d_ref_) the recorded intensity at an arbitrary reference distance. For each intensity I (d) for this same target at a distance d, the calibrated intensity, I_c_ (which is now independent of distance) is calculated as:

(6) $$I_{c}=\frac{I(d)}{f_{99\%}(d)+c_{\text{ref}}}*I(d_{\text{ref}})$$

To know the quality of the distance correction, a Root Mean Square Error (RMSE) between the value of the piecewise polynomial interpolation f_99%_ and the corrected intensity is calculated for each distance. Finally, as the value f(d_ref_) is unknown, the distance effect on the intensity is corrected with the following formula [Figure 3(II)]:

(7) $$I_{c}=f_{99\%}(d_{\text{ref}})+c$$

with c = f_99%_ (d) − I (d).

Further investigation on the intensity correction, reflectance relationship and radiometric calibration could be done. In [50], the author defines a backscattering coefficient related to the intensity in the case that the angle of incidence with the scanned surface is unknown. In [28], the authors define a logarithmic correction to estimate the reflectance value of a scanned object placed perpendicular to the TLS beam. They aim to be able to compare different TLS intensity. Unlike this paper, the logarithmic correction is not made because of the reliability of the materials used in this study. Therefore, we assume that the sensor of TLS does not change over time. Thus, a full radiometric calibrated intensity will be needed for future research.

#### 2.3.2. Determination of the Angle of Incidence with a Least Square Regression (LSR)

To obtain the angle of incidence with a surface (flat by assumption) represented by a selection from the point cloud, a Least Square Regression (LSR) is proceed on the sub-selection (Figure 3.III). As there are three different LSRs related to each vector of the XYZ-basis, the LSR is selected that minimizes the RMSE and allows at most 5% of the point cloud outside a pre-defined orthogonal distance d_i_ to the fitted plane. Finally, the normal angle to the plane is given by the coefficient of the plane equation. The angle of incidence ω with the surface equals:

(8) $$\omega =\left|\text{acos}\left(\frac{\vec{n.}\vec{x_{s}}}{\left\|\vec{n}\right\|\left\|\vec{x_{s}}\right\|}\right)-\pi \right|$$

with $\vec{x_{s}}$ the vector representing a reference point in the sub-selection and n⃗ the normal to the surface calculated by the LSR. It is remarkable that knowing the angle of incidence, one can have the normal to the surface as formally:

(9) $$\vec{n}=\{\begin{array}{l} \frac{\cos(\omega ).\left\|\vec{n}\right\|\left\|\vec{x_{s}}\right\|}{\vec{x_{s}}}\;\;\;\;\;\text{if acos}\left(\frac{\vec{n.}\vec{x_{s}}}{\left\|\vec{n}\right\|\left\|\vec{x_{s}}\right\|}\right)\in [k\pi ,(k+1)\pi ],\;\text{with k odd}\\ \;\;\;\;\;\;\;\;\;\;\;\;-\frac{\cos(\omega ).\left\|\vec{n}\right\|\left\|\vec{x_{s}}\right\|}{\vec{x_{s}}}\;\;\;\;\;\;\;\;\;\;\;\;\;\;\;\;\;\;\;\;\;\;\;\;\;\;\;\;\;\;\;\;\;\;\;\text{else} \end{array}$$

The accuracy and precision of this method is tested with the goniometric platform with increments of 10° of its azimuthal and elevation angles. As statistical indicators, the r^2^, slope and intercept of a linear regression of the angle determined by the LSR and the goniometric platform angle are given. The targeted platform is placed at approximately 2.05 m from the TLS beam aperture. Knowing the angle of incidence provided by the LSR, it can finally be related to the intensity averaged over the cloud of points selected (Figure 3.IV).

## 3. Results

### 3.1. Distance Effect Correction of the Intensity

**Figure 4.**
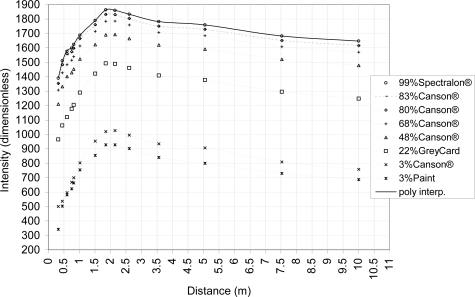
Intensity and distance relationship for the FARO LS880 for different materials placed perpendicularly to the laser beam.

As in [28], a vertical translation between the different intensity and distance relationships is revealed (Figure 4). Those translations have a very low standard deviation for materials with a reflectance value larger than 48% as presented in Table 2. This enables the generation of a reference piecewise polynomial interpolation on the 99% Spectralon® graph and to correct the distance effect on the intensity as discussed in point 2.3.1.

**Figure 5.**
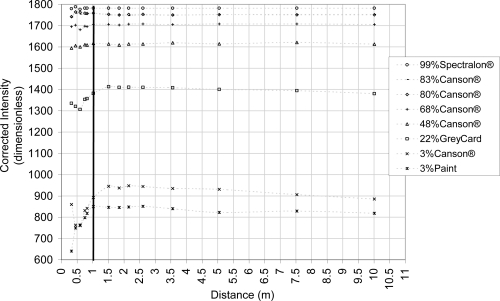
Correction of the distance effect on the intensity. The correction is valuable for distance greater than 1 m. The reference distance is 3.56 m.

Figure 5 shows the intensity correction given by the equation 6. With the equation notation, the reference distance used (d_ref_) is 3.56 m and f_99%_(d_ref_) = 1781.45 intensity units. The LS880 logarithm filter effect is clear as can show relationships between the various reflectances measurements. The distance effect correction with the piecewise polynomial is valuable for a distance larger than 1 m, especially for materials with a reflectance larger than 48%, while the 22%-reflectance Canson^®^ paper yields results of inferior quality. This result is analogous to the FARO LS HE80 used in [28]. The distance effect corrections of intensity value from materials with a reflectance of 3% (Canson^®^ and paint) have the worth quality and the graph shows unexpected differences in terms of reflectance that have not been detected by the spectroradiometer. Similarly, the difference between the 80% and the 83% Canson^®^ papers is not clear. The logarithmic correction suggested in [31] is not performed because of those two last reasons.

The RMSE between the translated 99%-spectralon piecewise polynomial interpolation used as reference at a distance of 3.56 m (Equation 6) and the corrected intensity is lower than 4 units (corrected intensity) for materials with a reflectance larger than 48%, whereas it is larger than 10 units (corrected intensity) for reflectance values smaller than 22% (Table 2).

### 3.2. Validation of the Angle of Incidence Provided by the Least Square Regression (LSR)

The angle of incidence provided by the LSR provides acceptable results with the goniometric platform. The regression of the correlation graph between the angle of incidence calculated manually and the one given by the LSR provides an r^2^ of 1, a slope of 1 for both horizontal and vertical rotation and an intercept of 1° for vertical and 2.8° for horizontal rotation.

### 3.3. Relationship between the Intensity and the Angle of Incidence for Pear Tree Leaves

#### 3.3.1. Establishing the Intensity and Angle of Incidence Relationship with Leaves on the Goniometric Platform

**Figure 6.**
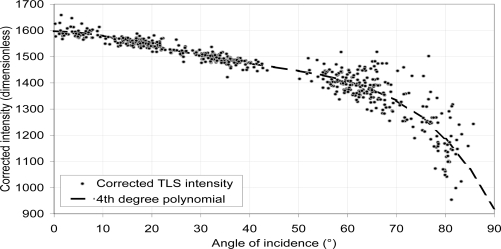
Corrected intensity and angle of incidence relationship for pear tree leaves placed on the goniometric platform. (•) is the average intensity of the selected point cloud representing the leaf. A fourth degree polynomial fitting is made to model this relationship (bold line). The angles of incidence is found by the LSR on the selected point cloud.

The angles of incidence provided by the LSR approximate the ones given by the protractors of the goniometric platform. Figure 6 shows that the angles of incidence vary with a maximum amplitude of ±10° around the angle of incidence measured manually. At 50°, there is a shift of +10° in the angle of incidence provided by the LSR. Indeed, despite the strings that are flattening the leaves, it is difficult to avoid wrinkling leaves when attached to the goniometric platform. In this way, the angles of incidence provided by the LSR are more realistic than the manually measured ones. Thus, those one are substituted by the angle of incidence provided by the LSR. Figure 6 shows that the intensity values increases with the decrease of the angle of incidence. In a first step, it increases quickly (+150 units for 10°) for angle of incidence decreasing from 85° to 55° and it starts to level off (+30 units for 10°) for angle of incidence decreasing from 55° to 0°. In addition, the variations of the intensity values are larger for angle of incidence larger than 55° (±100 units) whereas they are smaller for angle of incidence smaller than 55° (±30 units).

No clear difference appears between the azimuthal rotation of the goniometric platform and the elevation rotation. Given with a resolution of 5°, the curve of relationship between the corrected intensity and angle of incidence for the two different rotations are similar and the maximal absolute difference for the intensity is 20 units (corrected intensity) for an angle of incidence of 10°. This is negligible compared to the intensity variation as a function of angle of incidence. We get similar results in the comparison of the abaxial and adaxial sides of the leaves where the maximal absolute intensity difference 38 units (corrected intensity) for an angle of incidence of 10°, which is also negligible. Because of those two results, both cases are not taken into account in this study (graphs not shown).

**Figure 7.**
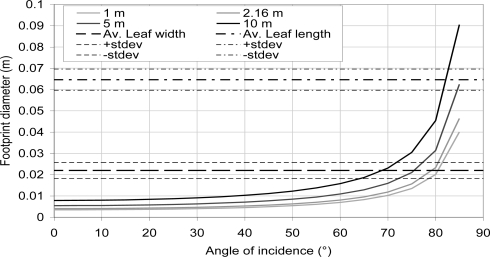
TLS beam footprint diameter as a function of angle of incidence and distance. This beam footprint diameter is compared to the average leaf widths and lengths.

Because of the size of the beam diameter and divergence, its footprint diameter could become larger than the leaf itself. It ranges from 0.046 m for an angle of incidence of 85° to 0.004 m for an angle of incidence of 0°. Figure 7 shows the variation of the footprint diameter as a function of angle and for the distance of 1, 2.16, 5 and 10 m. The diameter is calculated using Equation 4. Those footprint diameters are compared to the average widths and lengths of half pear tree leaves that were picked for the experiment. So, depending on the angle of incidence, the leaf size and the distance, the intensity values for the leaf material could be more sensitive to surrounding material. In Figure 6, the impact of the goniometric platform appears clearly for large angle of incidence. At this range (2.16 m) the intensity values decrease could be explained by the mixing of the goniometric platform and the leaves intensities.

At this distance, the TLS beam footprint diameter is 20% of the leaf width for an angle of incidence smaller than 20°, it is 45% of the leaf width for an angle of incidence greater than 65° and it exceeds the leaf width for an angle of incidence greater than 80°. Though the type of the laser sensor is unknown, the weight of the goniometric platform intensity could be lower than suggested in Figure 7 and the previous discussion, especially if it is Gaussian as discussed in [51]. In addition, the LSR may present some issues to accurately represent a surface with a large angle of incidence because the point cloud quality is worse at those angle of incidence compared to the one of a surface that is perpendicular to the beam [36].

In conclusion, retrieving the angle of incidence with the intensity would have a precision of ±5° and because of the diameter of the TLS beam footprint, it is not possible to measure the angle of incidence with the intensity for angle larger than 55–60°.

At a first sight, a logarithmic or cosine fitting could be made as it is insinuated in Equation 3 (b_1_ and b_2_). The intensity and angle of incidence relationship can be expressed as:

(10) log(ρ(ω).cos(ω))

As one can see, three functions appear:

- the logarithmic function that has not been corrected,
- a cosine function, and
- the reflectance value as a function of angle of incidence with the leaf surface ρ(ω).

As the optical properties of the leaves are unknown (they are not Lambertian [54]) and the logarithmic correction [28] cannot be made because their value of the TLS intensity for different reflectances are not consistent, as for example for low reflectance material (see Section 3.1.), a fourth order polynomial fitting is finally made on the relationship between intensity and angle of incidence.

#### 3.3.2. Testing the Relationship between the Corrected Intensity and the Angle of Incidence on *in-situ* Pear Tree Leaves

**Figure 8.**
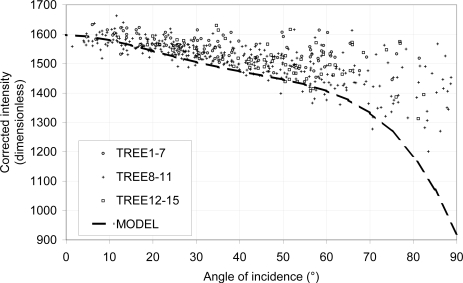
Test of relationship between intensity and angle of incidence for leaves of *in-situ* pear trees. The bold line represents the reference curve established with the leaves on the goniometric platform.

The test shows a vertical and positive translation in the intensity values for angles smaller than 60° (Figures 8) compared to the previously established relationship. It presents more variability. There is no clear difference between the different distances intensities, which means that the distance correction is valid.

As in the previous experiment, the intensity increases with an angle of incidence decrease, but the measured intensity values are higher. It could be interpreted in two ways: (i) it is higher in terms of intensity and is vertically translated to +50 units (corrected intensity) or (ii) it is larger in terms of angle of incidence and is horizontally translated to +10°.

In addition, the precision to find an angle of incidence from the corrected intensity for angles of incidence smaller than 60° is larger than in the previous experiment: (i) ±10° for angles of incidence smaller than 30° and (ii) ±15° for angles of incidence ranging from 60° to 30°.

Many reasons could occur to explain those two facts:

- **Curvatures:** It is possible that in the selection, undesirable parts of the point cloud are selected. Their intensity have a varying impact in the average intensity depending on the quantity of these undesirable points, whereas those points can easily have an impact in the LSR and thus on the angle of incidence. For instance:
  - if a leaf that is perpendicular to the beam is selected and if this selection includes a sub-selection which forms a plane which is almost parallel to the beam, then, the LSR on this selection will provide an angle of incidence larger than expected and with a higher intensity (depending on the quantity of undesired points that are selected). That would be the reason why Figure 8 presents only a few selections with an angle of incidence smaller than 5°.
  - With a similar reasoning, the selection of a leaf including a zone which has a large angle of incidence with the TLS beam and a curved zone could present a smaller angle of incidence than expected.
- **Wrinkles:** In the case where a leaf that has many wrinkling is selected, then the impact of these on the intensity is significant whereas the LSR will not consider them.
- **Footprint and point cloud quality:** As the leaves are not placed onto on a larger flat surface such as the goniometric platform, the quality of the point cloud representing those leaves is lower especially for large angle of incidence and for increasing distances. This is the reason why there is a lack of data for the two groups of trees 1–7 and 12–15, especially for angle of incidences ranging from 90° to 60° (Figure 8 and Table 3). As in [35], an increase of the angle of incidence implies a decrease in the point cloud precision.
- **Footprint and intensity mixing:** As in point 3.3.1, the footprint has a great impact on the intensity, especially for angles of incidence larger than 60° (Figure 7). This may be the reason why the data are different for those angles as the surrounding scene is different. However, the intensity values should be more accurate than the ones provided by the measurement on the goniometric platform, especially for angles of incidence smaller than 60° and despite the decrease in precision.
  **Physiology:** The scans did not proceed at the same time. There is an 8 days difference between the scans with the leaves placed on the goniometric platform and the scans of the trees.
  **Multiple scattering:** The scans proceeded under different conditions than in the case of leaves placed on the goniometric platform. Because of the complexity of the canopy, it is possible that a multiple scattering effect occurs which results in a higher than expected intensity [52].
  **TLS radiometric calibration**: Due to the fact that the TLS has not been entirely calibrated, it is possible that the intensity of low reflectance objects changed through time. Nevertheless, the scans having 8 days difference, one might expect that the sensitivity of the sensor has not moved as in [28].

#### 3.3.3. Testing the Corrected Intensity and Angle of Incidence Relationship on an Entire Leaf

**Figure 9.**
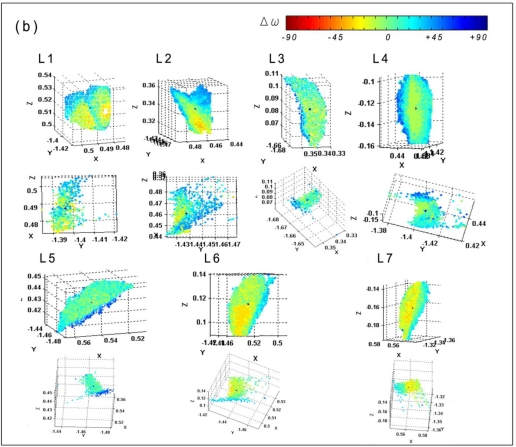

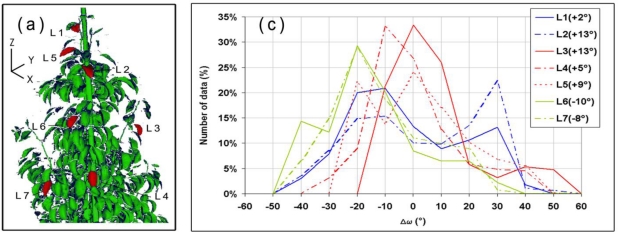
**(a)** Selection of seven leaves on tree n°9; **(b)** The figures shows the leaves as they appear to the TLS (up) and their side view (down). Δ ω is plotted (Colors). X, Y and Z are the points coordinates in the scan (m); **(c)** Distribution (normalized) of Δ ω and difference between the angle of incidence provided by the LSR on the entire leaf and the intensity VS angle of incidence relationship (caption). Three groups are emphasized depending on the shape of the distribution: (blue) two peaks, (red) centered but stopped at ∼+20°, (green) positive shift.

Figure 9(a) shows a selection of seven entire leaves on tree n°9 (see also Figure 3). For those selections, ghost points are mostly retained. A LSR is made on those entire leaves and provides a reference angle of incidence that is subsequently compared to the angle of incidence provided by the average intensity for each point of the selected leaves (see Figure 6). The differences Δω (equation 5) are plotted for each point [Figure 9(b)]. The fitted plane lies on the points that have a Δω equal to zero (green). If Δω tends to be yellow or red, then it means that the leaf tends to face the beam compared to the LSR angle of incidence, whereas if it is blue or purple, the leaf tends to be on the side. The distributions (quantity normalized) of the Δω are shown [Figure 9(c)]. In addition, the difference between the LSR angle of incidence and the one provided by the average intensity is given [Figure 9(c), inset]. Those differences could have an uncertainty of +10 to +15° because of the presence of a vertical translation in the intensity and angle of incidence graph as it has been previously discussed.

Some of the assumptions of the previous section are confirmed by these measurements:

- **Curvatures:** It appears that leaves with a simple curvature (similar to a cylinder) as leaves n° 3 to 7, have a skewed normal distribution for their Δω. For leaves n° 3 to 5, this distribution is translated to respectively + 13°, + 5° and + 9°. It means that their average intensity represents well the angle of incidence provided by the LSR as it has been previously seen.
  At the opposite, the average intensity for leaves n° 6 and 7 is translated to respectively −10° and −8°. For leaf n° 6 it could be explained by the case i.a) of the Section 3.3.2. as the side of the leaf forms a large angle of incidence with the beam. This provides a larger angle of incidence [Figure 9 (b)] which is not balanced by the low intensity of this set of point in the average intensity [Figure 9(c)].
- **Wrinkles:** Leaves n° 1 and n° 2 illustrate well the impact of wrinkles on the intensity. Both show a multimodal distribution.
- **Footprint and point cloud quality:** In general, ghost points are presents for surface with large angle of incidence as it is also shown by the other leaves. Thus, one can consider that a point with a low intensity has a higher probability to be a ghost point. Leaf n° 2 shows a large surface that has a large angle of incidence with the beam but those points does not look like ghost points. In fact, this leaf seems to be lengthened. In this case, it would be more efficient not to delete those point, but to correct them, depending on their positions on the leaf.
- **Footprint and intensity mixing, physiology, multiple scattering and TLS radiometric calibration:** those factors are not tested since the scan used in this last study is the same than in the previous one.

## 4. Discussion

In this study, Conference pear tree leaves are scanned and the intensity data provided by the TLS is analyzed with a particular focus on its properties for describing geometry of leaves. Prior to that, the intensity is corrected for the distance effect and the angle of incidence provided by the LSR is tested on the goniometric platform. Then the relationship between the corrected intensity and the angle of incidence is determined with flattened leaves placed on a goniometric platform. Next, this relationship is tested on flat part of the leaves that are still attached to the pear trees. Finally, the angle of incidence is determined using a LSR on an entire leaf, and this notwithstanding leaf curvatures and wrinkles. The Δω (see equation 5) is mapped on the selection to understand the impact of those curvatures and of those wrinkles on the data. To summarize, the three set-ups of measurement are resulting in a LSR for four different conditions:

- for the goniometric platform only,
- for flattened leaves placed on the goniometric platform,
- for some parts of the leaves that are fixed on the tree,
- for entire leaves that are fixed on the tree.

It appears that the flattened leaves on goniometric platform provide a good precision (±5°) but maybe a poor accuracy in terms of finding the angle of incidence with the intensity and because of the incomplete radiometric calibration of the TLS intensity. In the case where this radiometric calibration is sufficient, the test made on the partial selection of leaves on the tree would provide a more accurate result (+50 units of corrected intensity). Still, this last test brings a lower precision in the definition of the relationship (from ±10° to ±15° depending on the angle of incidence). This shift in the accuracy could be explained either by:

- the LSR conditions (a low RMSE with a limited number of points that are away of the LSR plane) and the leaf curvatures and its wrinkles,
- the impact of the footprint diameter of the TLS beam,
- the physiological state of the plant, the radiometric calibration of the TLS or even a multiple scattering occurring in the canopy.

In the last test, it is clear that wrinkles and undulations are playing a large role in the precision. It is also shown that angles of incidence larger than 60° with pear tree leaves will provide bad results in term of accuracy and precision. Even the measurement on the goniometric platform could not provide better information because of the 3%-reflectance painting surrounding the leaves and so in the mixing of their intensity in the point cloud. As previously seen, scanning larger leaves could reduce this angle of incidence limit. In addition, if the second experiment shows a consistency in the distance correction, it appears that distance plays a great role in the capacity to extract a good point cloud and this to make a LSR with enough points. That probably depends on leaf size, and one might expect that the measurements should be extended to a wider range and with larger leaves. In general, distance, angle of incidence and leaves dimensions should be taken into account for the set-ups of scanning that aim at extracting leaf geometry. The measurement set-ups suggested in [20] could be improved in this way.

In addition, it would be also recommended to test the relationship between intensity and angle of incidence for trees with flat leaves to study the multi scattering effect and/or to change the conditions of selection for the LSR set in point 2.3.2. In the future, a complete radiometric calibration should be set to guaranty the consistency of accuracy of the relationship.

Notwithstanding the aforementioned issues, different potential uses for the intensity can be envisaged. First, the third experiment emphasized the fact that intensity could help in determining the points having a higher probability to be a ghost point:

- The points with a low intensity have a higher probability to be ghost points because they are on the part of the leaves having a large angle of incidence. Those points could be directly deleted or corrected.
- In the case where points with a low intensity constitute a large zone on the leaf, it is more difficult to determine whether they are ghost points or not. This zone appears larger than in the reality. In conclusion, closer is the point to the leaf border, higher is the probability that this one is a ghost point. In those cases, the points should be only corrected as their deletion would diminish the size of the leaf.

Those two points could be used to eliminate ghost point and view as an improvement of the pre-processing methods for point cloud (as e.g., [57]). In addition, the intensity could help to extract the angle of incidence to the leaf and thus, the normal of the leaf surface as shown in the equation 9. Viewed then as a map of Gauss on the surface “leaf” [55], these intensities could be enough to rebuild the leaves from a “simplified” point cloud.

Alternatively, using the difference between the angle of incidence provided by the LSR and comparing it to the incidence angle provided by the intensity for each point of the selected leaves (Δω) would give an estimation of the curving and the wrinkling of the leaves and this thanks to the Δω distribution. This could be used as a wrinkle indicator as the amplitude of those ones might be not large enough compared to the distance precision of the TLS. Finally, a promising future in the use of the intensity is given by its use as representing the normal of the surface of leaves.

Finally, new opportunities exist to use the intensity to detect physiological aspects of the leaf such as the chlorophyll content with a LEICA ScanStation2 (532 nm) [49]. Lastly, combining different wavelengths of TLS laser beams, one would get information on the physiological status of vegetation as it has been done with hyperspectral measurements [53]. This would also help to understand the spread of diseases and stress within the canopy.

## 5. Conclusions

We have investigated the properties of intensity in relation to distance and angle of incidence with leaf surfaces. The distance effect on intensity has been corrected to set a constituent relationship between the intensity and the angle of incidence. The variation of the intensity through angle of incidence seems to be a good indicator to help in the extraction of leaves geometries from TLS point cloud.

Results show that one can expect a precision of ±5° to derive the angle of incidence from intensity data in the case of flat leaves. The results with curved leaves have clearly shown that the curvatures and the wrinkles are the reason for the degradation of the precision in the relation between the intensity and the angle of incidence. Therefore, we could expect to use the intensity to determine angle of incidence with a precision of ±5°.

Two general applications are emphasized in this study:

- Knowing the size, orientation and distance of the leaves, the scanning set-up can be improved for intensity and point cloud quality.
- Intensity could help eliminating/correcting the ghost points, it may help to derive the surface of the leaves and it could be translate to an indicator (Δω distribution) helping to know the geometry of the leaves (wrinkles, curves).

Finally, the corrected intensity could be used to reduce the impact on the point cloud of the factors (i)–(vi) of the introduction.

## Figures and Tables

**Table 1.** Feature provided by the TLS FARO LS 880 constructor.

**Measurement principle**	Continuous wave phase shift

**Field Of View (vertical x horizontal)**	320° × 360°
**Wavelength**	785 nm (Near Infra-Red)
**Diameter beam aperture**	3 mm, circular
**Beam divergence**	0.014°
**Sensor FOV**	3 mrad
**Angle resolution used in this publication**	0.018°
**Range**	0.6 m–76 m
**System distance error (Accuracy)**	±3 mm at 25 m

** Repeatability at 10 m (Precision)**	**(RMS for filtered / raw data)**

**90 % reflectance**	0.7/2.6 mm
**10% reflectance**	1.3/5.2 mm

**Table 2.** (i) Vertical translation (average on the distance) between the intensity value of the 99%-Spectralon® and the intensity value of other materials, (iii) Raw value at the reference distance (3.56 m), (iv) RMSE between the interpolation function f_99%_ of the 99%-Spectralon® intensity (minus a constant, at the reference distance) and the measured intensity for distance larger than 1 m. Raw values range between 0 and 2047.

	**(i) Shift average (Raw value)**	**(ii) Standard deviation (Raw value)**	**(iii) Raw value at 3.56m**	**(iv) RMSE (corrected intensity)**

**83%Canson®**	30.24	3.90	1749	2.98
**80%Canson®**	27.84	4.88	1750	2.50
**68%Canson®**	79.07	5.76	1705	1.60
**48%Canson®**	169.55	5.36	1619	3.66
**22%GreyCard**	399.30	29.87	1408	11.49
**3%Canson®**	884.93	49.66	839	21.52
**3%Paint**	961.55	34.09	935	11.49

**Table 3.** Distances of the point cloud sub-selections for each of the 15 trees and their number of sub-selections that have been made for the LSR plane fitting. The increase of the distance increases the difficulty to make a correct LSR (no extraction is possible for tree n°15). Trees are grouped by their distances to the beam aperture (>1 m).

**Tree n°**	**Min. dist. (m)**	**Max. dist. (m)**	**# data**

1–5	2.92	4.30	26
6	2.43	3.22	21
7	1.83	2.52	50
8	1.55	1.99	100
9	1.44	1.83	100
10	1.32	1.94	70
11	1.36	1.96	70
12	1.68	2.55	30
13	2.15	3.06	24
14	3.02	3.15	4
15	X	X	X
